# Lessons From the Edge of Reactivation: Managing Herpes Simplex Virus Encephalitis Years After Initial Infection in High-Risk Neurosurgical Patients

**DOI:** 10.7759/cureus.77883

**Published:** 2025-01-23

**Authors:** Takashi Matsumori, Ichiro Takumi, Takashi Asahi, Kyoko Tatebayashi, Hidetoshi Murata

**Affiliations:** 1 Department of Neurosurgery, St. Marianna University School of Medicine, Kawasaki, JPN; 2 Department of Neurosurgery, Kanazawa Neurosurgery Hospital, Nonoichi, JPN; 3 Department of Neurosurgery, Mitsui Memorial Hospital, Tokyo, JPN

**Keywords:** antiviral therapy, corpus callosotomy, epilepsy surgery, herpes simplex virus encephalitis, postoperative complications

## Abstract

Herpes simplex encephalitis (HSE) is a severe central nervous system infection with significant morbidity and mortality. While rare, reactivation of HSE following neurosurgical procedures is increasingly reported, particularly in patients with a previously clinically significant HSE, which is the most critical risk factor for reactivation. This case highlights the challenges of managing HSE reactivation risk in pediatric patients with a history of HSE, who are considered high risk due to their susceptibility to viral reactivation. As demonstrated in a nine-year-old girl with intractable epilepsy who underwent corpus callosotomy, postoperative day seven was marked by fever, altered consciousness, and seizures. Delayed antiviral therapy led to severe neurological sequelae. Identified risk factors included perioperative steroid use and surgical stress, with the history of HSE standing out as the predominant risk factor. This case underscores the importance of defining high-risk groups, specifically patients with a prior history of HSE, and proactively managing their care, including consideration of prophylactic antiviral therapy. Early recognition, timely intervention, and comprehensive perioperative strategies are essential to mitigating the risk of severe outcomes in this vulnerable population.

## Introduction

Herpes simplex encephalitis (HSE) is a severe central nervous system infection caused by herpes simplex virus (HSV), associated with significant morbidity and mortality [1]. It has been reported that 8% of patients succumb to the condition, while 69% experience long-term neurological sequelae [1]. Early diagnosis and prompt antiviral therapy, particularly with acyclovir, are critical for improving survival and neurological outcomes [2]. However, early recognition can be challenging due to the nonspecific nature of initial symptoms, especially in pediatric patients.

HSE reactivation has been increasingly reported in patients with a history of HSE undergoing neurosurgical procedures [3], particularly highly invasive epilepsy surgeries [4,5]. Factors such as perioperative stress, surgical manipulation, and steroid use are thought to contribute to the risk of reactivation [2].

In this report, we present a rare case of HSE reactivation in a pediatric patient with intractable epilepsy secondary to prior HSE, following corpus callosotomy. This case underscores the complexities of managing high-risk postoperative patients and highlights the critical importance of early recognition and timely intervention in HSE reactivation.

## Case presentation

Patient background

A nine-year-old girl, who had been diagnosed with HSE at the age of four, was treated with antiviral therapy during the acute phase of the illness. Although she experienced persistent brain atrophy, particularly in the left insular cortex and mesial temporal region, she initially exhibited no notable motor impairments.

**Figure 1:**
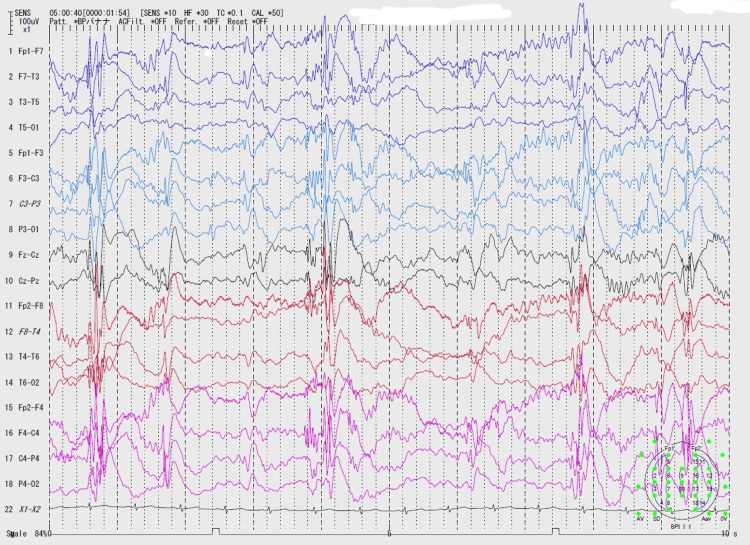
The results of the EEG examination in the preoperative evaluation are shown EEG: electroencephalography This recording is in stage II sleep, where multifocal polyspikes and spindles are observed. The EEG test display is based on the international 10-20 method, and each montage is shown on the vertical axis. This test was displayed in a bipolar montage. Also see the upper left for the filtering conditions and scale

**Figure 2:**
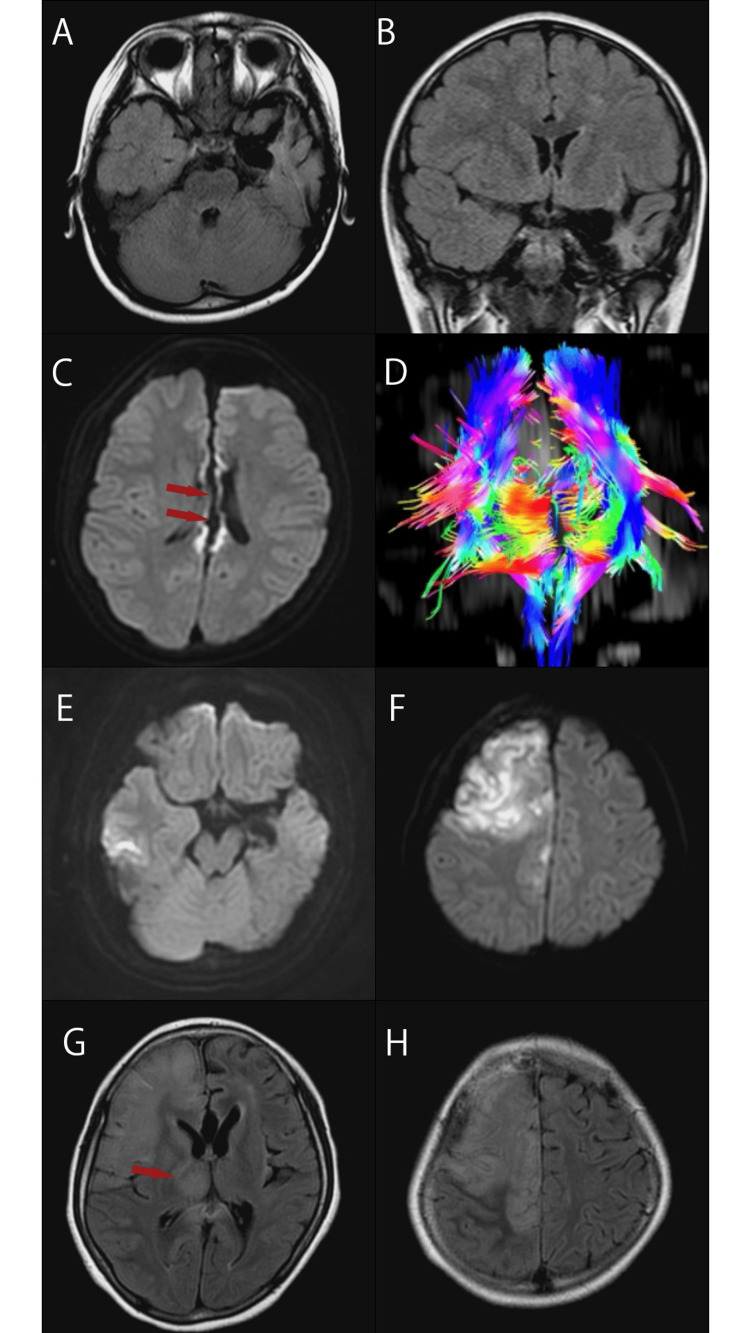
MR images are shown in chronological order MR: magnetic resonance; POD: postoperative day; FLAIR: fluid-attenuated inversion recovery After the initial HSE, lesions remained from the lateral left temporal lobe to the insular cortex (A: axial FLAIR image, B: coronal FLAIR image). Post-corpus callosotomy MR images show minimal impact on surrounding areas (C: axial diffusion image, arrowhead; D: tractography by diffusion tensor imaging). The MR image on POD 10 shows no abnormalities in the initial lesion site of the left temporal lobe (E: axial diffusion image), but a lesion is observed as a hyperintense area in the right frontal lobe (F: axial diffusion image). On the MR image of POD 23, the lesion has expanded to the entire frontal lobe (G: axial FLAIR image, H: axial FLAIR image), and it is evident that the lesion extends to the thalamus (arrowhead)

At the age of seven, she began to suffer from epileptic seizures. These included focal motor seizures, focal to bilateral tonic-clonic seizures, or sometimes atonic falls. Despite being treated sequentially with various antiepileptic drugs, carbamazepine, levetiracetam, zonisamide, lamotrigine, valproic acid, and clobazam, her seizures could not be adequately controlled, even under the careful management of a pediatric neurologist and pediatric epilepsy specialist. Electroencephalography (EEG) revealed generalized spike-and-slow-wave discharges at a frequency of 1-1.5 Hz (Figure 1). Magnetic resonance imaging (MRI) indicated significant left hemispheric brain atrophy extending from the insular cortex to the mesial temporal region (Figures 2A, 2B). At the age of eight, her intellectual abilities were assessed, and her intellectual quotient (IQ) was found to be between 45 and 55 by Wechsler Intelligence Scale for Children-Fourth Edition.

By the age of nine, she was experiencing daily seizures, including focal motor seizures with bilateral tonic extension and atonic seizures occurring two to five times per day. Diagnosed with intractable epilepsy, she was referred to the epilepsy center at St. Marianna University School of Medicine for further evaluation and management.

Preoperative evaluation and surgical procedure

Video EEG confirmed epileptic seizures but failed to identify a clear focal onset. To achieve better seizure control, a complete corpus callosotomy was planned. A complete corpus callosotomy was performed without intraoperative complications (Figures 2C, 2D). Postoperatively, the patient developed laryngeal edema following extubation, necessitating reintubation. She was successfully extubated later the same day after receiving intravenous hydrocortisone (200 mg).

Postoperative course

After surgery, the patient developed a fever of 38.0°C between the second and fourth postoperative days (POD 2-4), which was resolved by POD four. On POD seven, her condition worsened with a high fever of 40.0°C, pharyngeal erythema, tonsillar swelling, and altered consciousness. Laboratory tests showed mild inflammatory markers, and she was managed conservatively at first.

By POD nine, her fever and altered consciousness persisted, necessitating mechanical ventilation. On POD 10, she developed involuntary movements in her left upper limb. Diffusion-weighted imaging (DWI) MRI showed hyperintensity in the right frontal lobe (Figures 2E, 2F), suggesting possible new brain involvement. Cerebrospinal fluid (CSF) analysis revealed a cell count of 66/μL, predominantly mononuclear cells (Table 1). The CSF sample underwent polymerase chain reaction (PCR) testing for HSV DNA, but the results were not yet available at that time.

**Table 1:** Cerebrospinal fluid test results performed on POD 10 and POD 30 C: counts; WBC: white blood cell; PCR: polymerase chain reaction; POD: postoperative day Quantitative testing for herpes simplex virus (HSV) DNA was conducted using quantitative PCR. Aerobic, anaerobic, and enrichment cultures were negative on both POD 10 and POD 30

	POD 10	POD 30	Reference value
Appearance	Clear	Clear	
WBC count	66	39	5/μL
Mononuclear cell count	<1	1	
Specific gravity	1.005	1.006	1.005~1.007
pH	8.0	8.0	7.31~7.34
Occult blood	+	Trace	-
Total protein	36	76	10~50 mg/dL
Glucose (serum glucose level)	57 (118)	63 (101)	50-80 mg/dL
HSV DNA quantification	90 x 10	<100	<100 C /mL

Her condition was provisionally diagnosed as febrile status epilepticus. However, as her symptoms showed no improvement over the following three days, intravenous acyclovir treatment was initiated at a dose of 30 mg/kg/day on POD 14, prior to the confirmation of HSV in the CSF. On POD 17, PCR results confirmed the presence of HSV DNA in the CSF, leading to a diagnosis of HSE (Table 1). This acyclovir treatment continued for three weeks, during which follow-up CSF analysis confirmed the absence of HSV DNA, indicating successful viral suppression. On POD 23, MRI revealed a progression of hyperintensity in the right frontal lobe (Figures 2G, 2H).

Subsequent clinical course

By the time of discharge, seizure frequency had decreased to 2-3 episodes per week. However, the patient’s IQ declined to approximately 30, and severe left hemiparesis persisted, with an MMT score of 1/V for the upper limb and 2/V for the lower limb. She was transferred to a rehabilitation facility on POD 94. As of 85 months postsurgery, the patient is currently being treated with valproic acid, phenytoin, perampanel, lamotrigine, and lacosamide under the care of a pediatric epilepsy specialist and continues to experience focal motor seizures with bilateral tonic extension approximately twice per week and focal impaired awareness seizures lasting several seconds three times per week. Severe left hemiparesis persists, requiring the use of short-leg orthoses and a wheelchair.

## Discussion

This case highlights the rare but serious complication of HSE reactivation following neurosurgical procedures in a patient with a history of HSE at age four and subsequent epilepsy at age seven. On POD seven, the patient developed fever, altered consciousness, and seizures, with imaging and CSF analysis confirming HSV DNA positivity by POD 17. Although antiviral therapy was initiated, delayed treatment resulted in severe sequelae, including left hemiparesis and cognitive decline. The recurrence of fever on POD seven, coinciding with neurological symptoms, aligns with previous reports where MRI findings, particularly limbic lesions, were pivotal for diagnosis. Perioperative steroid use, frequently implicated in such cases, likely contributed to viral reactivation.

Postoperative fever is commonly observed following neurosurgery [6], with primary causes ranging from surgical site infections to inflammatory responses. Fever exceeding 38.5°C has been reported in 43% of pediatric epilepsy surgery cases within the first 12 postoperative days [7], particularly in procedures such as hemispherectomy and multilobar resection, which are strongly associated with febrile episodes due to ventricular opening during surgery. Such fever may lead to delays in the initiation of antiviral therapy.

A review of HSV encephalitis cases following neurosurgical procedures highlights the diverse contexts in which this complication can occur [3], affecting patients of all ages and across various types of surgeries. The interval between symptom onset and diagnosis ranges from a few hours to as long as three weeks, with MRI findings not always exhibiting the characteristic temporal lobe lesions associated with HSV encephalitis. Steroid use was a notable factor, documented in the majority of cases, and 30% of patients had a prior history of HSV infection. The average time from surgery to symptom onset was calculated to be approximately 7.7 days. HSV encephalitis has been reported not only after craniotomy but also following other procedures such as coil embolization [8] and cardiac surgery [9], demonstrating its occurrence in a broad range of surgical settings.

Integrating our case with the 11 reported instances of HSE reactivation following epilepsy surgery [4], it is notable that four patients received perioperative steroids [9-11], with symptoms manifesting, on average, 9.7 years after the initial HSV infection. The prognostic outcomes were concerning: only five cases achieved full recovery, whereas seven resulted in significant neurological sequelae or mortality [4]. Although HSV-1 is predominantly implicated, documented cases of HSV-2 further underscore the pathogenic variability of this condition [12].

Prophylactic antiviral therapy has been proposed as a strategy to reduce the risk of HSE reactivation, particularly in patients with a history of HSV infection [2,3,10]. In the presence of even minimal suspicion of HSE reactivation, immediate CSF analysis should be performed alongside the initiation of antiviral therapy without awaiting definitive results. Notably, studies have reported that up to 24% of cases may initially yield negative CSF findings when analyzed within the first three days of symptom onset [11], underscoring the importance of clinical vigilance and prompt intervention. Given that antiviral medications are generally well-tolerated and associated with relatively few complications, this approach offers a feasible option for high-risk patients with a previous history of HSE. Empirical antiviral therapy should not be discontinued based solely on negative PCR results if clinical suspicion remains high. Although rare, HSE reactivation has been reported even during prophylactic antiviral therapy [12]. In cases where antiviral therapy proves insufficient, immunosuppressive therapy may be effective [13].

## Conclusions

Patients with a history of HSE are at high risk of reactivation during epilepsy surgery or other neurosurgical interventions. Vigilance for postoperative fever or neurological changes is critical. Early symptom recognition, prompt antiviral therapy, and thorough perioperative management are essential to prevent severe outcomes. Prophylactic antiviral therapy should be strongly considered in this high-risk group. Clear protocols for identifying and managing these patients are vital to improving outcomes and ensuring effective preventive and therapeutic measures.
